# Age-associated global DNA hypermethylation augments the sensitivity of hearts towards ischemia-reperfusion injury

**DOI:** 10.3389/fgene.2022.995887

**Published:** 2022-11-15

**Authors:** Sri Rahavi Boovarahan, Nemat Ali, Abdullah F. AlAsmari, Alaa Alnoor Alameen, Rehan Khan, Gino A. Kurian

**Affiliations:** ^1^ Vascular Biology Lab, School of Chemical and Biotechnology, SASTRA Deemed University, Thanjavur, India; ^2^ Department of Pharmacology and Toxicology, College of Pharmacy, King Saud University, Riyadh, Saudi Arabia; ^3^ Department Department of Pathology, Case Western Reserve University, Cleveland, OH, United States

**Keywords:** ageing, ischemia reperfusion injury, mitochondria, mRNA expression, DNA methylation

## Abstract

Most pre-clinical studies in cardiac ischemia-reperfusion injury (I/R) are carried out in young or old animals, which does not cater to the adult age in humans who encounter I/R. Not many studies in the literature are available that emphasize the sensitivity of the adult heart to injury from the young heart, where there exist distinct alterations in DNA methylation and mitochondrial function that contribute to injury. In the present study, we utilized young (8 weeks old) and adult (24 weeks old) rat hearts to evaluate distinct DNA methylation alterations that contribute to I/R injury. The cardiac basal physiological activities in young and adult rat hearts were insignificantly changed from normal. But the DNA hypermethylation and expression level of mitochondrial genes were slightly higher in adult rat hearts. The consequential effect of these changes was measured in the I/R heart to understand its response to additional stress. Accordingly, we noted an increase in global DNA hypermethylation levels by 40% and 62% in young and adult I/R hearts, respectively, from their respective control. Subsequently, a decline in mitochondrial genes (ND1, ND4L, ND6, Cyt B, COX1, COX2, and ATP8) that regulate cardiac contractility was observed in adult I/R hearts. These changes, in turn, reduced hemodynamics (Rate pressure product) by 51% and 32% in adult and young I/R hearts, respectively, from their controls. Besides, the I/R-linked infarct size was higher in adult hearts (58%) than in young hearts (37%). Correlation analysis showed a significant negative correlation of global DNA methylation with the MT-ND1 expression (r = −0.7591), MFN2 expression (r = −0.8561) and cardiac RPP (r = −0.8015) in adult I/R hearts. Based on the above observations, we concluded that age promoted DNA methylation and deteriorated cardiac responsive ability to resist I/R injury.

## 1 Introduction

Ischemic heart disease (IHD) is still a clinical burden that accounts for the highest proportion of morbidities and mortalities under non-communicable diseases, despite many scientific advancements in this field (Khan et al., 2020). Although numerous studies have underlined the mechanism of IHD in various pre-clinical models, the translation of these scientific findings from pre-clinical trials to the clinical side often is partially successful. This is because certain critical parameters associated with IHD patients, like co-morbidities or progressing age, are often overlooked in the pre-clinical model. The prevalence rate of IHD in the middle-aged/adult human population has been increasing in recent years and has reached an alarming state. However, the majority of existing pre-clinical research in rats did not account for the distinct physiology of the aging process and is heavily dependent on too young (8–12 weeks) rats or included old (48 weeks) animal models (Jackson et al., 2017). The young and aged animals exhibit significant differences in physiology, basic disease biology, cardiac tolerance, drug efficacy and its mechanism that may affect the study outcome. Besides, the young age group is undergoing developmental processes with a specific gene expression pattern, and the aged group may be entering the senescence phase (Jackson et al., 2017). So ideally, animals with 16–24 weeks of age may be reliable to account for the adult human population, as the onset of disease and development of the co-morbidities initiate at this stage.

Recent studies have shown that aging not only alters the cardiac structure and function during IHD but also increases the vulnerability of cardiac tissue to withstand additional stress (Dai et al., 2012), that include ischemia reperfusion injury (I/R). I/R is an unavoidable injury that occurs during the revascularization of the ischemic area of IHD patients’ hearts upon CABG and PCI surgical treatments (Kalogeris et al., 2012). Advanced age was reported to be associated with elevated susceptibility to myocardial injury after ischemia/reperfusion, primarily due to decreased coronary circulation and collateral flow (Azhar et al., 1999). Unlike young hearts, aged hearts exhibit structural changes like stiffness, increased left ventricular wall thickness and fibrosis (Dai et al., 2012); and functional changes like diminished response to increased workload and change in hemodynamics and signaling (Isoyama and Nitta-Komatsubara, 2002). Further, aging induces a defect in the repair mechanism and thereby increases the sensitivity of the heart towards disease prevalence like IHD (Strait and Lakatta, 2012). Most of the physiological adaptive changes associated with the aging process in the heart negatively impact the cardiac response to ischemia reperfusion (Liu et al., 2012).

Incidentally, no effective therapy is available till now to completely attenuate I/R injury, and the scenario is critical in adult and aged hearts subjected to revascularization procedures. The phenotypic change in an aging heart starts at the gene expression level, which is regulated by epigenetic modifications. Importantly, one of the factors that contribute to I/R pathology is the altered epigenetic regulation of cardiac genes (Wang et al., 2021). However, no studies are available in the literature that connects the consequence of epigenetic modifications in basal tissue of the adult heart that contribute to the I/R pathology.

Cardiac aging is associated with the complex interplay of DNA methylation and histone posttranslational modifications, characterized by decreased heart functions and ventricular and atrial remodeling (Zhang et al., 2018). These epigenetic modifications control the transcript of RNA products, which regulate gene transcription, chromatin remodeling, and mRNA processing and modifications. Among the different epigenetic modifications, histone acetylation and deacetylation have been demonstrated to be involved in the pathology of I/R (Wang et al., 2021). Inhibition of HDAC conferred cardioprotection when it was given during reperfusion (Xie et al., 2014). Like HDAC, DNA methylation can control the transcriptional metabolic program in the heart and modify the metabolic response to different cellular environmental stress, including age-induced cellular changes. In fact, DNA methylation’s contributory role in cardiac I/R is well established and was used as a potential target (Boovarahan et al., 2022; Boovarahan and Kurian, 2022).

Another well-understood key mediator of I/R injury is mitochondrial dysfunction, where the normal functioning of the organelle can be controlled by epigenetic regulation. Cardiac tissue is enriched with mitochondria because of its high energy demand. In turn, it can regulate epigenetic control by providing the intermediate metabolites necessary to generate and modify epigenetic marks in the nucleus (Matilainen et al., 2017). Interestingly, this, in turn, can regulate the expression of mitochondrial proteins, as the majority of these proteins are encoded by nuclear genes (Matilainen et al., 2017). Many studies have demonstrated that the normal aging process is linked with a decline in mitochondrial quality and activity (Sun et al., 2016). Mitochondrial dysfunction is one of the significant hallmarks of aging, and mitochondrial preservation is vital for the effective recovery of the heart from I/R. The majority of reports in the literature demonstrated that aging-induced elevated mitochondrial DNA mutation impaired the mitochondrial replication control gene POLGA (Moore et al., 2020), declined the mitochondrial bioenergetics capacity and increased the ROS production (Moore et al., 2020), signifying the strong influence of aging on mitochondria.

On the whole, the I/R heart is characterized to have the presence of elevated oxidative stress and perturbed mitochondria, where epigenetic modulation also contributes negatively to the pathology (Boovarahan and Kurian, 2021; Boovarahan and Kurian, 2022). However, most of the I/R-linked cardiac pathological cellular changes is overlapped with the cellular level changes associated with age progression. Interestingly, most of the studies on cardiac I/R in pre-clinical studies were on young rats (8–12 weeks old), which does not cater to the adult age-induced changes in the heart. Only very few studies are available in the literature exploring the sensitivity of the adult heart to I/R injury from the young heart. Therefore, understanding how the heart responds to the cumulative effect of age-linked and I/R-associated epigenetic changes when subjected to I/R is critical and remains unknown. In the present study, we evaluated the cardiac response of young and adult rat hearts to I/R injury from the perception of DNA methylation, one of the key epigenetic modifiers in I/R.

## 2 Materials and methods

### 2.1. Animals

All procedures involving the animals were reviewed and approved by Institutional Animal Ethics Committee (IAEC), SASTRA University, Thanjavur, India (CPCSEA Approval No. 300/SASTRA/IAEC/RPP/) and were conducted in accordance with the CPCSEA (Committee for the Purpose of Control and Supervision of Experiments on Animals) guidelines. Animals were housed in polycarbonate cages and maintained at 25 ± 2°C with 12 h light/dark cycle and relative humidity of 65 ± 2%. Feed and water were provided *ad libitum*.

### 2.2 Experimental groups

The animals were divided randomly into four experimental groups 1) 8 weeks old young control (N), 2) 8 weeks old I/R (I/R); 3) 24 weeks old adult control (A_N); 4) 24 weeks old adult I/R (A_I/R) on a random basis (*n* = 6/group). The rats were anesthetized with sodium thiopentone (60 mg/kg i. p.). The hearts were excised, mounted on a Langendorff apparatus and treated as per the experimental groups. Briefly, the rats in the normal groups (N and A_N) were perfused for 120 min with KH buffer (NaCl−118.0 mM, KCl−4.7 mM, CaCl2−1.9 mM, MgSO4−1.2 mM, NaHCO3−25.0 mM, KH2PO4−1.2 mM and glucose−10.1 mM). In contrast, the I/R group rat hearts (I/R and A_I/R) were perfused with KH buffer for 30 min (stabilization period) and then stopped for 30 min (to induce global ischemia), followed by KH buffer perfusion to the heart for 60 min (to induce reperfusion).

### 2.3 I/R injury assessment

I/R injury was assessed in terms of cardiac recovery by monitoring the hemodynamic changes in the left ventricle using LabChart Pro eight software and Power Lab Data Acquisition System (AD Instruments). A latex balloon (connected to a pressure transducer) was inserted inside the left ventricle, and the left ventricular pressure changes (LVP) were recorded. The LVP data was further utilized to determine the changes in heart rate, left ventricular developed pressure (LVDP), and maximum and minimum pressure derivative (dp/dt). The rate pressure product (RPP), the measure of cardiac recovery, was calculated by multiplying LVDP and heart rate. The cardiac injury was further confirmed by infarct size measurement using TTC stain (Ferrera et al., 2009). Briefly, the heart sections were stained with 1.5% Tetrazolium Chloride (TTC), washed with PBS (pH:7.4) and fixed with formaldehyde solution. The heart sections were then imaged. ImageJ software (NIH-USA) was used to analyze the infarct area.

### 2.4 DNA methylation analysis

DNA was isolated from the heart tissues using Phenol/Chloroform/Isoamyl alcohol method, followed by ethanol precipitation (Yeung, 2002). Global DNA methylation was assessed using MethylFlash™ Global DNA Methylation (5-mC) ELISA Easy Kit (Epigentek) with the isolated DNA samples. The DNMT enzyme activity was then evaluated in the cardiac nuclear extract using the EpiQuik™ DNMT (DNA Methyltransferase) Activity/Inhibition Assay Ultra Kit.

### 2.5 Gene expression analysis

Total RNA was extracted from rat heart samples using TRIzol reagent, followed by the cDNA conversion using the cDNA synthesis kit protocol (Thermo Fisher Scientific, MA, United States). The mRNA expression of the genes was evaluated using real-time PCR analysis with DyNAmo Flash SYBR (Thermo Fisher Scientific) in system ABI 7500 thermal cycler system (Applied Biosystems, CA, United States). The mRNA expression of the genes contributing to DNA methylation (DNMT1, DNMT3A, DNMT3B, TET1, TET2, and TET3), hypertrophy (ANP, BNP), mitochondrial proliferation and regulation (PGC-1α, POLGA, TFAM), mitofission ((MFN1, MFN2), mitofusion (MFF, FIS, DNM1), autophagy (PINK1, PARKIN, OPTN), mitochondrial complex I activity (ND1, ND2, ND3, ND4, ND4L, ND5, ND6), complex III activity (Cyt B), complex IV activity (COX1, COX2, COX3); complex V activity (ATP6, ATP8) were assessed using the real-time PCR analysis with the isolated mRNA samples. The primer sequences of the genes are given in Supplementary Table S1. The gene expression was normalized using the expression of GAPDH in the samples. The relative gene expression was further calculated by the method of Livak et al. (Livak and Schmittgen, 2001). Mitochondrial DNA copy number (Mt DNA copy number) was estimated as the ratio of relative gene expression of Mt-ND1 gene to nuclear-encoded beta-actin gene expression in DNA samples. The primer details are provided in Supplementary Table S1.

### 2.6 Mitochondrial isolation and evaluation of mitochondrial functional activities

Mitochondria were isolated by density-gradient separation from the homogenized heart tissue as per the protocol mentioned in Palmer et al. (Palmer et al., 1977). Briefly, cardiac tissues were homogenized in the buffer containing 100 mM KCl, 40 mM Tris HCl: pH 7.5, 10 mM tris base, 40 mM MgCl_2_, 1 mM EDTA, 1 mM ATP in a proportion of 10 ml/g heart. The tissue homogenate (10%) was subjected to density-gradient centrifugation at 4°C, 600 g for 10 min. Later, the supernatant was collected and centrifuged further at 6,000 g, 4°C for 10 min. The obtained mitochondrial pellet was then purified by dissolving it in the aforementioned mitochondrial isolation buffer containing bovine serum albumin 1.5% and centrifuged for 10 min at 12000 g to yield a pure mitochondrial pellet. Finally, the mitochondrial pellet was dissolved in a storage buffer. The mitochondrial protein concentration was estimated using Bradford reagent (BioRad) with bovine serum albumin as a standard. Mitochondrial ATP content was determined using ATP lite (Perkin Elmer) according to the manufacturer’s instructions. Mitochondrial electron transport chain (ETC) enzyme activities were measured in the isolated mitochondria using a spectrophotometer. Briefly, the mitochondria were supplemented with specific complexes’ donors and acceptors, and monitored for the changes in enzyme activity across groups, as described previously (Barrientos et al., 2009).

### 2.7 Statistical analysis

All data were represented as the mean ± SD. The significance level between the groups was assessed with a one-way ANOVA test followed by Dunnett’s post hoc analysis using Graph Pad Prism 7.0 software. Correlation analysis was performed using the Pearson coefficient method.

## 3 Results

### 3.1 Adult rat hearts exacerbate the I/R associated infarct size than young rat hearts

**FIGURE 1:**
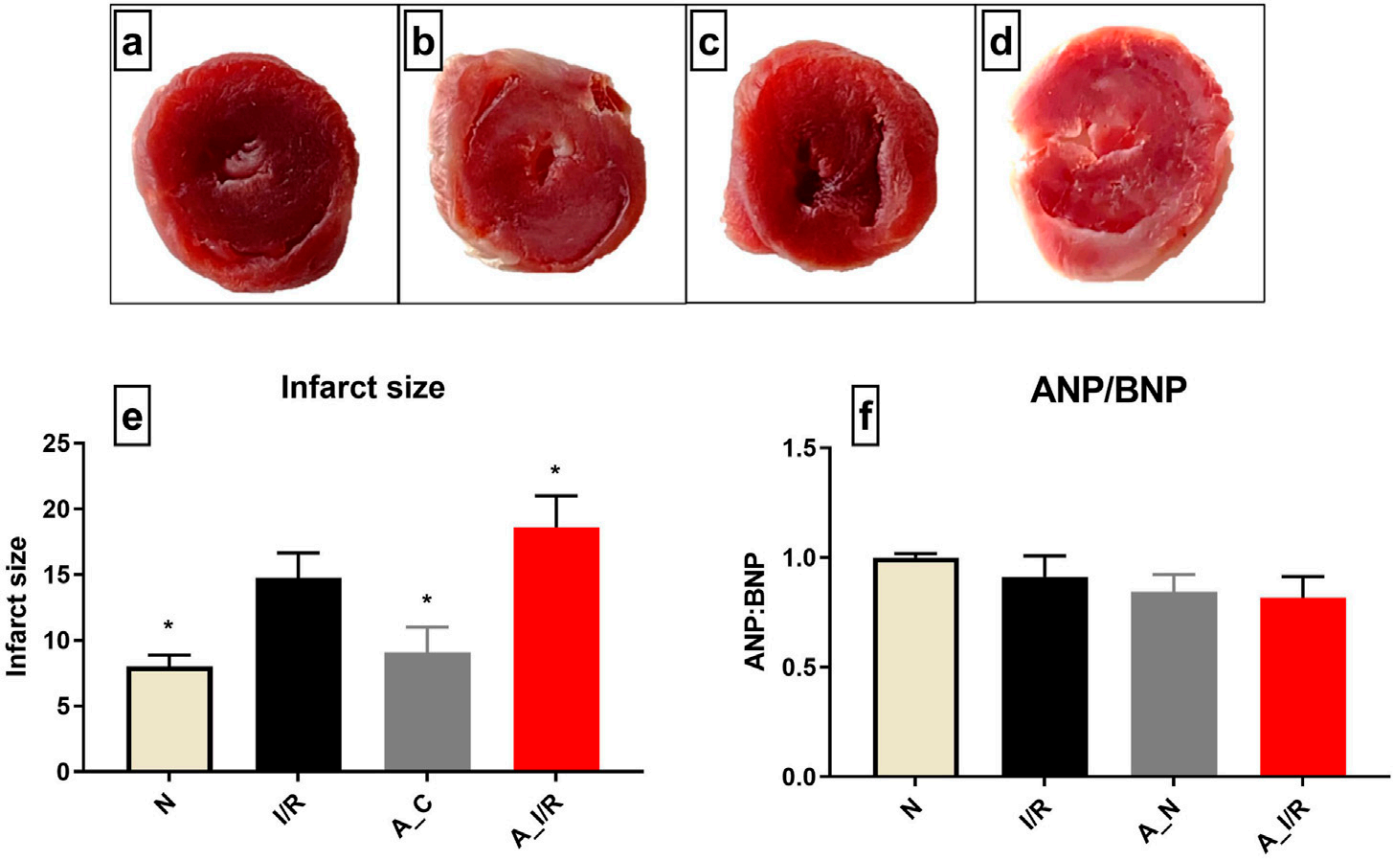
Cardiac I/R injury assessment in young and adult rat hearts: The representative TTC stained images of isolated rat hearts subjected to the perfusion protocol as per the groups: **(A)** N, **(B)** I/R, **(C)** A_N, **(D)** A_I/R. **(E)** represents the percentage of infarct size. The representative images were obtained at ×10 magnification. **(F)** represents the ANP/BNP ratio. The graph represents the mean ± SEM. *p < 0.05 vs. *I/R*.

According to Figure 1, no visible changes in the cardiac architecture were noted at a basal level between the young and adult sham rat hearts. The I/R-challenged young rat hearts exhibited an increased infarct size by 37% from the young sham heart, while the adult I/R hearts showed a further increase in infarct size by 58% from the young sham hearts. However, no significant changes in the ANP/BNP ratio were observed in the young and adult I/R hearts.

### 3.2 Adult rat hearts exhibited more prominent alterations in hemodynamic indices than young rat hearts subjected to I/R

Adult rat hearts at basal level showed a decline in cardiac performance, measured *via* RPP (18%), +dp/dt (13%), -dp/dt (17%) when compared with the young rat hearts (Table 1), but the changes were insignificant. I/R injury in young hearts exhibited a significant decline in the cardiac hemodynamic parameters RPP, +dp/dt and -dp/dt by 32%, 41%, and 43%, respectively, when compared to the young sham hearts. In the case of adult hearts, ischemia-reperfusion highly compromised the cardiac function, evident by a further decline in RPP and rate of relaxation and contraction by 51%, 54%, and 55%, respectively, from young sham rats’ hearts. However, the I/R-induced hemodynamic changes were similar in both I/R and A_I/R hearts compared to their respective sham.

**TABLE 1:** Impact of I/R on cardiac hemodynamic indices in young and adult rat hearts: The impact of I/R was evaluated by the hemodynamic changes LVDP (left ventricular diastolic pressure); RPP (Rate Pressure Product = heart rate × LVDP) and ±dp/dt (maximum/minimum force of contraction). The data are represented as mean ± SEM. *p < 0.05 vs. *I/R*.

Groups	LVDP (x10 mmHg)	RPP(mmHg*beats/min*10^4)	(-Dp/dt) (x10 mmHg)	(+dp/dt) (x10 mmHg)
N	9.1 ± 1.2^*^	2.4 ± 0.24^*^	69.3 ± 8.7^*^	94.6 ± 10.3^*^
I/R	4.9 ± 0.5	1.6 ± 0.1	33.9 ± 3.9	45.6 ± 5.8
A_C	7.0 ± 1.4^*^	1.8 ± 0.19^*^	61.2 ± 6.7^*^	78.7 ± 9.9^*^
A_I/R	3.2 ± 1.1^*^	1.2 ± 0.13^*^	31.1 ± 3.7^*^	42.4 ± 5.3^*^

### 3.3 Global DNA methylation levels differ in young and adult rat hearts, contributing to the I/R pathology

**FIGURE 2:**
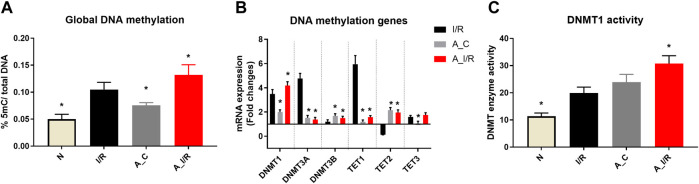
Methylation changes in I/R challenged young and adult rat hearts: The global level changes in methylation were assessed from **(A)** global DNA methylation level; **(B)** The gene expression of DNA methylation enzymes (expressed as fold changes from normal); and **(C)** DNMT enzyme activity. The graphs represent mean ± SD values. *p < 0.05 vs. *I/R*.

The basal tissue level global DNA methylation changes of adult and young sham hearts were measured and the results are given in Figure 2. Adult sham hearts exhibited a global DNA hypermethylation by 28% from young sham hearts at the basal level. The corresponding significant increase was noted in DNMT1, 3A and 3B mRNA expression by 1.9, 1.5 and 1.7 folds, respectively. DNMT activity, measured by the ELISA technique, showed relatively increased activity in adult sham hearts compared to young sham hearts. Among the TETs, only TET2 expression was increased in adult sham hearts, while TET1 and TET3 did not show any significant changes from the young sham hearts.

I/R in young hearts exhibited hypermethylation of DNA at the global level by 40%, along with a significant increase in mRNA expression of DNMTs, TET1 and TET3 (Figure 2) in coherence with our previous reports. But in adult rat hearts, I/R induced elevation in the global DNA methylation level was 62%, with subsequent increase in DNMT1 expression and activity by 76% and 63%, respectively, from young sham hearts.

When we considered I/R-specific methylation changes without considering the basal changes, the extent of methylation induced by I/R in young and adult hearts was similar. Adult I/R hearts showed a 46% and 23% increase in global DNA methylation level and DNMT1 expression from adult sham. However, unlike young I/R rat hearts, adult I/R hearts did not show significant changes in the DNMT3A and DNMT3B expression from the respective sham.

Correlation analysis showed a significant negative correlation of global DNA methylation with the cardiac RPP (r = −0.8015), indicating the influence of global DNA methylation and cardiac physiology (Table 2).

**TABLE 2:** Correlation data of global DNA methylation with the injury and mitochondrial function during cardiac I/R in adult rats: Pearson correlation of global DNA methylation with the cardiac hemodynamic parameters and listed mitochondrial genes has been calculated and the corresponding *p*-value and r-value are presented. *p < 0.05* was considered statistically significant.

Parameter	Correlation coefficient r-value	*p*-value
RPP	**−0.8015**	**0.0489**
Gene expression		
PGC 1α	**−**0.7322	0.0791
Dnm1	**−**0.3789	0.1382
Parkin	**−**0.3023	0.1294
MFN1	**−**0.6204	0.3891
MFN2	**−0.8561**	**0.0466**
DRP1	**−**0.4927	0.1782
MFF	**−**0.5792	0.1936
FIS	**−**0.5489	0.1845
PINK1	**−**0.4903	0.2895
TFAM	**−**0.4555	0.0954
POLG1	**−**0.4901	0.0856
ND1	**−0.7591**	**0.048**
CYTB	**−**0.482	0.1732
ND6	**−**0.5639	0.1378
ND5	**−**0.7904	0.1789
ND4L	**−**0.5609	0.1263
ND3	**−**0.4932	0.1696
COX3	**−**0.6492	0.1882
ATP6	**−**0.4946	0.1783
ATP8	**−**0.8043	0.0793
COX2	**−**0.5598	0.0853
COX1	**−**0.7823	0.1289
ND2	**−**0.7689	0.1323
ND4	**−**0.6038	0.1947

The significant values have been highlighted for easy identification.

### 3.4 Expression of cardiac mitochondrial bioenergetics genes downregulated more in adult rat heart than in young rat heart

**FIGURE 3:**
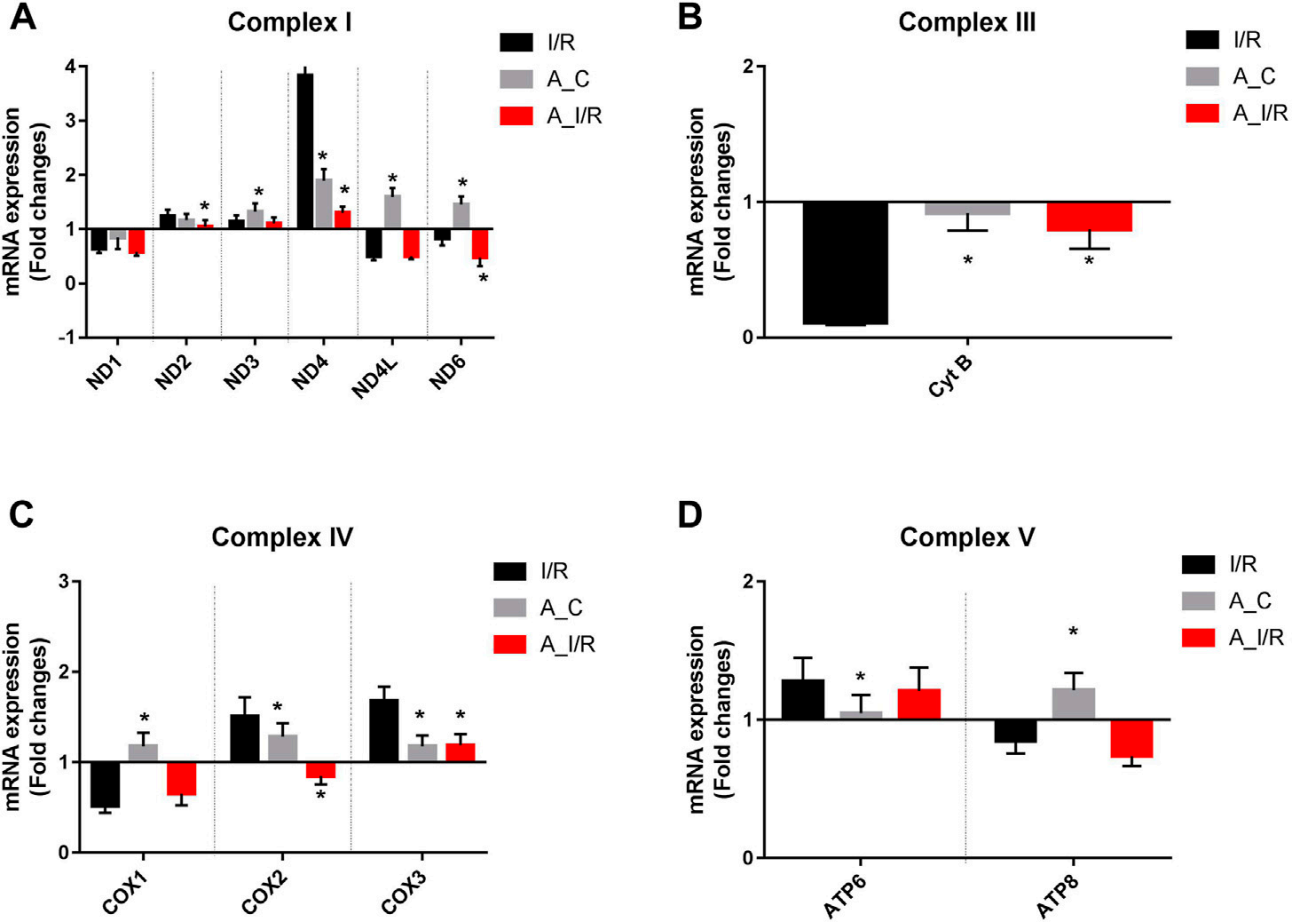
Differential expression of mitochondrial encoded ETC genes in young and adult hearts during I/R: The mRNA expression of the ETC genes encoded by mitochondrial are presented for **(A)** Complex I; **(B)** Complex III; **(C)** Complex IV; **(D)** Complex V. The values are expressed as fold changes from normal. The graphs represent mean ± SD values. *p < 0.05 vs. *I/R*.

Deteriorated mitochondrial bioenergetics function is one of the cardinal features in the event of both I/R pathology and the aging process. Therefore, we measured the mRNA expression of the mitochondrial encoded 13 ETC genes involved in mitochondrial bioenergetics. Adult sham rat hearts did not show any significant reduction in the expression of the 13 ETC genes from the young sham. Upon I/R insult, both young and adult hearts exhibited significant downregulation of ND1, ND6, CytB, COX1, and ATP8 genes with a higher degree of decline in adult I/R hearts when compared to the young sham hearts. Additionally, the adult I/R hearts showed a significant decline in the expression of ND4L and COX2 genes compared to the young and adult sham rat hearts (Figure 3).

**FIGURE 4:**
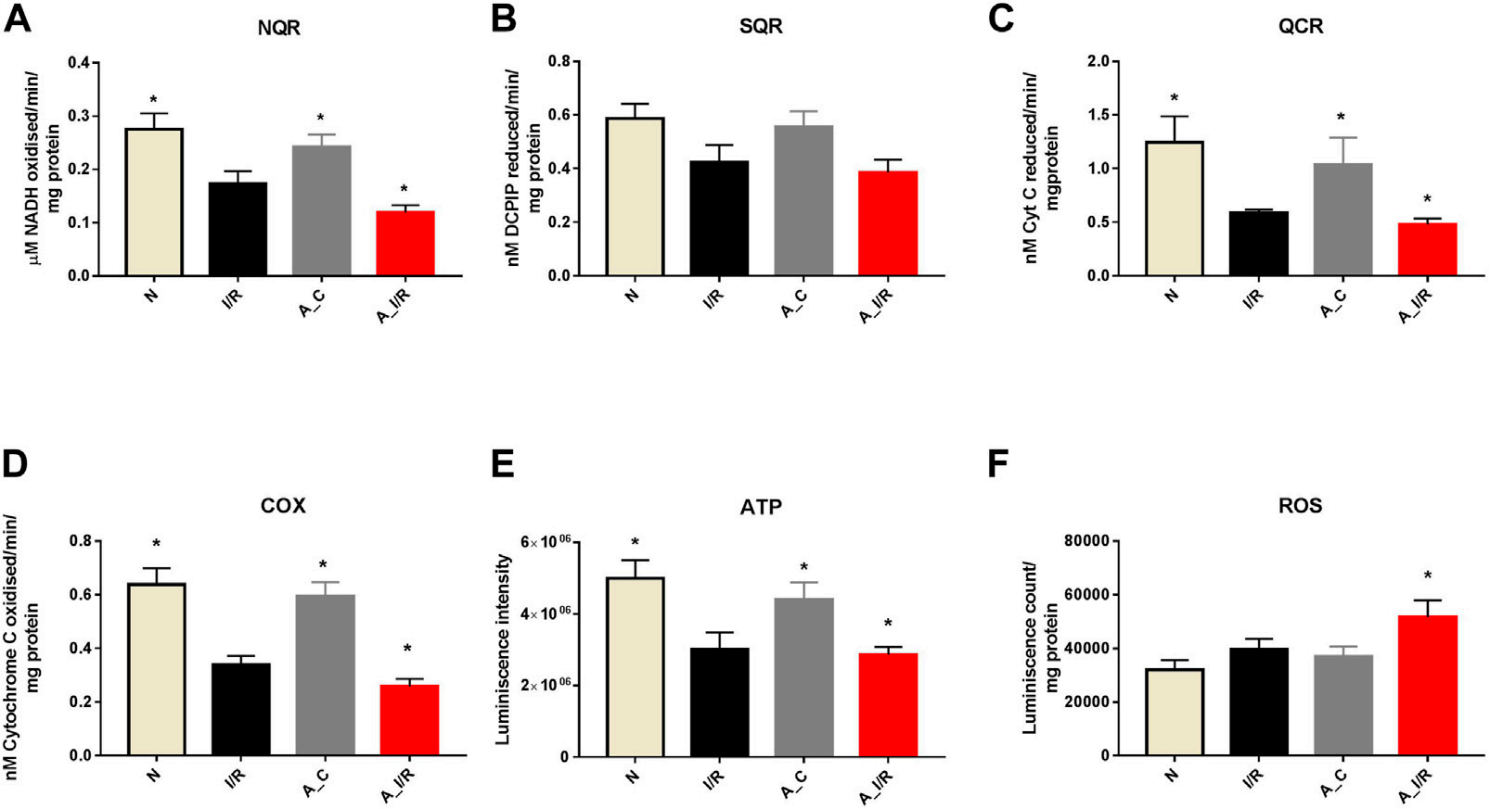
Assessment of mitochondrial function in young and adult I/R hearts: The impact of I/R on mitochondrial functional activities in young and adult hearts were assessed by evaluating the electron transport chain activities of **(A)** Complex I (NQR); **(B)** Complex II (SQR); **(C)** Complex III (QCR); and **(D)** Complex IV (COX); **(E)** ATP content; **(F)** ROS levels. The graphs represent mean ± SD values. *p < 0.05 vs. *I/R*.

To further check the impact of the changes in ETC gene expression in the heart, we measured the ETC enzyme activities in the young and adult I/R hearts. At the basal level, the adult sham hearts did not show significant changes in the ETC activity, ATP and ROS levels compared with the young sham. The downregulation of the ETC genes in I/R hearts reflected in the ETC activities as well, where the complex activities I, III and IV were declined by 37%, 53% and 50% in young I/R hearts and by 52%, 61% and 58% respectively in adult I/R hearts when compared with a young sham rat heart. I/R also decreased the overall ATP levels and increased the ROS level in both young I/R and adult I/R hearts when compared with the young sham (Figure 4).

Correlation analysis of the mRNA expression of the ETC genes with global DNA methylation in the aged I/R hearts showed a strong negative correlation of DNA methylation with MT-ND1 expression (r = −0.7591, *p* = 0.0480) (Table 2).

### 3.5 Genes involved in mitochondrial replication, fission, fusion and mitophagy were significantly downregulated in adult I/R rat hearts than in young I/R rat heart

**FIGURE 5:**
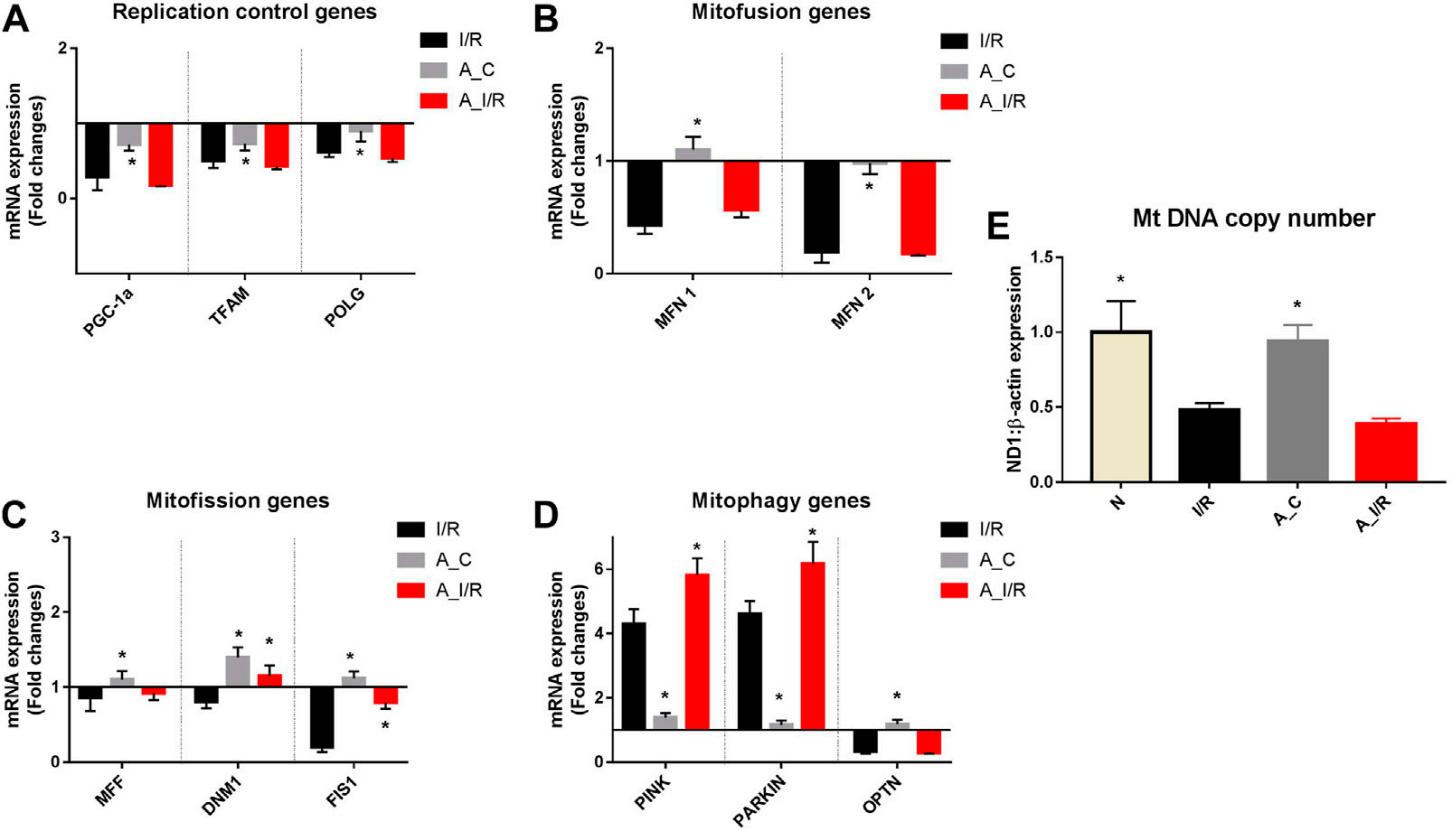
mRNA expression analysis of mitochondrial dynamics genes in I/R-challenged young and adult hearts: The mRNA expression of the genes involved in the processes of **(A)** Mitochondrial replication; **(B)** Mitofusion; **(C)** Mitofission; and **(D)** Mitophagy are presented. The values are expressed as fold changes from normal. **(E)** represents the end effector mitochondrial DNA copy number. The graphs represent mean ± SD values. *p < 0.05 vs. *I/R*.

Figure 5 shows differential mitochondrial quality control gene expression patterns (mitochondrial replication, mitochondrial fission, fusion and autophagy) of young and adult I/R rat hearts in comparison with their sham rat heart. Among the three genes screened for mitochondrial replication assessment, only PGC-1α and TFAM significantly downregulated at basal level in adult rat hearts compared to young hearts. But in the I/R heart, all these genes, PGC-1α, TFAM and POLGA, were significantly downregulated in both young and adult rat hearts, with a higher degree of downregulation in PGC-1α and TFAM genes in adult I/R hearts (0.18 and 0.43 folds respectively from young sham (Figure 5A).

Further, we assessed the genes in the mitofission and mitofusion process as they maintain the healthy mitochondrial copy number post-replication. Both the mitofusion genes MFN1, MFN2, and mitofission genes MFF and FIS1 did not exhibit any significant changes in their expression in the adult sham compared with the young sham. However, upon I/R, both the mitofusion genes MFN1 and MFN2 were significantly downregulated in both young and adult hearts from the young sham. Among the mitofission genes, only Fis1 showed a decline in young I/R and adult I/R hearts compared to young sham and their respective shams.

We analyzed the mRNA expression of the genes responsible for mitophagy as they maintain the quality of functional mitochondria. No significant differences were observed in the expression of these genes between adult sham and young sham hearts. I/R increased both PINK and PARKIN mRNA expression by 4.3 and 4.5 folds in young I/R hearts, respectively, which further increased to 5.8 and 6.2 folds, respectively, in adult I/R rat hearts when compared to the young sham rat hearts. In fact, I/R-induced upregulation in PINK and PARKIN genes in adult hearts was notably significant from the adult sham hearts.

The mitochondrial DNA copy number, determined by the changes in this mitochondrial quality gene expression, was not altered in the adult sham when compared with the young sham hearts. I/R decreased the mitochondrial DNA copy number significantly by 52% and 62% in young and adult I/R rat hearts compared to the young sham. However, the changes in the copy number between adult sham and adult I/R were insignificant.

To understand the influence of methylation on the quality control genes in adult I/R hearts, we made a correlation study and found that the expression levels of the MFN2 gene significantly correlated negatively with the DNA methylation (Pearson correlation coefficient r value = −0.8561) (Table 2).

## 4 Discussion

Recent studies have shown that ageing not only alters the cardiac structure and function but also increases the vulnerability of cardiac tissue to stress-induced abnormalities (Dai et al., 2012). This is mainly because of the genetic alterations in their gene expression mediated by epigenetic control in different stages of the aging process and the influence of associated environmental factors (Herman et al., 2021). Accordingly, transient expression and inhibition of genes change the different physiological and metabolic events of young and adult hearts. A recent study has emphasized that healthy adult hearts experienced increased susceptibility and vulnerability to cardiac stress and injury than young hearts (Dai et al., 2012). But the subcellular changes that contribute to the enhanced sensitivity in adult healthy heart to additional cardiac stress is not well explored and addressed in the present study. Our results showed that the cardiac response to ischemia-reperfusion differs significantly between young and adult hearts, despite both sets of hearts being physiologically healthy at the basal level. This finding suggested that age-related subcellular changes may be responsible for the distinct behavior of the heart due to the coordinated action of different cell organelles involved in normal cardiac physiology. Nevertheless, we found higher infarct size in the heart along with deteriorated hemodynamic parameters from ischemia-reperfusion-challenged young rat heart.

One of the major subcellular determinants involved in cell injury and contractile dysfunction is mitochondrial function (Anzell et al., 2018). According to the present study data, mitochondrial dysfunction associated with I/R was more prominent in adult rat hearts than in young rat hearts. This was in agreement with a higher degree of I/R altered expression of cardiac mitochondrial genes like ND1, ND4L, ND6, CytB, COX1, COX2 and ATP8, which were significantly highly expressed in a healthy adult heart. Since the gene expression is controlled transiently by epigenetic modification at DNA and histones level, we measured global DNA methylation, which plays a critical role in the regulation of mitochondrial genes, where few genes are mitochondrial DNA encoded and histones are absent in mitochondrial DNA. Our results showed significantly higher global DNA hypermethylation in the adult heart than young I/R heart. But when we consider basal level methylation, I/R-associated global DNA hypermethylation was similar between young and adult rat hearts indicating that the basal tissue changes are a key determinant factor in I/R injury in adult hearts.

Aging can affect the structure and function of the heart and is characterized to have prolonged contraction and relaxation time in the cardiomyocytes (Fleg and Strait, 2012). The basal level difference in the isolated young and adult hearts with respect to hemodynamics was suggestive of an alteration in the cardiac response. An autonomic imbalance between the sympathetic and parasympathetic nervous systems can compromise cardiac stability and function (Floras and Ponikowski, 2015). By using an isolated rat heart model to assess the cardiac contractility, where the neuro-hormonal influence on cardiac function was absent, we could distinguish the inherent ability of cardiac response in the young and adult heart. According to the result shown in Table 1, the contractile efficiency of the adult heart was insignificantly reduced from the young heart, that in turn, affects the overall performance of the heart from an ischemic attack. Many investigators have shown a reduced age-related cardiac responsiveness to cardio-stimulatory signalling pathways like adrenergic receptors (Ferrera et al., 2009). Similarly, investigators have also demonstrated distinct drug responsiveness in cardiac tissue with prolonged age (Mangoni and Jackson, 2004). Even though many investigators are aware of these distinctive differences between the myocardium in different age groups, many prefer young animals to study the disease pathology that generally affects the elderly. Interestingly, most myocardial infarction studies commonly conducted on rodents used ages of 8–12 weeks, equivalent to a human age of 20–25 years (Jackson et al., 2017). Few studies have shown that the onset of the disease in rodents generally occurs after 16 weeks. Similarly, co-morbidity development may happen in rodents after 16 weeks of age (Jackson et al., 2017). All these observations noted by different investigators emphasize that the role of aging is often overlooked in many existing animal studies and that demand new investigations.

In the present study, we identified a significant difference in the global DNA methylation level between young and adult rat hearts that may regulate differential gene expression. The corresponding DNMT1 expression was also higher in adult rat hearts than in young rat hearts. We analyzed the cardiac mitochondrial activity and the corresponding gene expression to understand whether these basal level changes were responsible for reducing the contractile function. However, no significant difference in the mitochondrial activity or gene expression was noted between young and adult rat heart, suggesting that hypermethylation of DNA adversely affect the expression of non-mitochondrial genes involved in the contractile function of the heart.

Next, we assessed whether the basal level changes in the DNA methylation reduced the responsiveness of the heart to additional stress incurred *via* ischemia-reperfusion. Both infarct size and compromised hemodynamics were high in the adult rat heart when compared with the young rat heart, indicating that cardiac responsiveness to the additional stress was altered in both hearts. Higher injury and low ischemia recovery of adult heart confirmed the existence of age-related changes in the adult heart that may stemmed at the cellular and molecular level. When we compared adult sham hearts with young sham hearts, a 10% decrease in PGC-1α expression was observed. Among the mitochondrial encoded bioenergetics genes, MT-ND6 and MT-CYT B were significantly downregulated in adult rat hearts than in young rat hearts. Interestingly, the gene expression of MT_NDL4 and MT-COX two was upregulated in young rat hearts, whereas in adult rats, these genes were downregulated significantly.

Similarly, the mitochondrial control genes like PGC-1α, POLGA and TFAM were also significantly downregulated in both groups, where a prominent decline was shown in adult rat hearts. The same pattern of changes was observed for the other replication control, mitofission, mitofusion and mitophagy gene expressions. This indicates that age-associated modification may play a critical role in low mitochondrial tolerance to the I/R injury. Mt DNA copy number, the end effector, determined by the changes in this mitochondrial quality gene expression, was decreased significantly by 52% in young I/R hearts, which escalated to a decrease by 62% in adult I/R rat hearts, probably contributing to a higher cardiac injury in adult hearts subjected to I/R.

The previous report in the literature showed DNA methylation plays a significant role in regulating bioenergetic genes like PGC-1α, ND1, ND6, Cyt B, COX2, and COX3 (Stoccoro and Coppedè, 2021). Accordingly, in the present study, DNA methylation was increased in I/R rat hearts with a corresponding increase in DNMT one activity and its gene expression in both young and adult old rats. Higher DNA methylation of a gene is reported to decrease the activity of the gene. Accordingly, we noted declined gene expression of these genes in cardiac I/R rat hearts from both young and adult old rats. These data confirmed that age-associated mitochondrial dysfunction and DNA methylation collectively contribute to I/R-associated injury. Further correlation analysis of the mRNA expression of these ETC genes with global DNA methylation in the aged I/R hearts showed a strong negative correlation of DNA methylation with MT-ND1 expression (r = −0.7591, *p* = 0.0480).

Based on the results obtained in the present study, we found that adult rat hearts induced hypermethylation of DNA and mitochondrial dysfunction by upregulating mitophagy and mitofission genes and downregulating mitochondrial replication, ETC and mitochondrial fusion genes even at the basal level. Upon I/R insult, the adult rat hearts showed a higher degree of methylation and mitochondrial dysfunction at gene, resulting in increased I/R injury than the young hearts. The I/R-associated changes were similar in young and adult I/R hearts. However, the pre-existing changes in DNA methylation and mitochondrial dysfunction in adult age contributed to its enhanced injury during I/R.

## Data Availability

The original contributions presented in the study are included in the article/Supplementary Materials, further inquiries can be directed to the corresponding authors.
