# Supplementary material for: Age-associated global DNA hypermethylation augments the sensitivity of hearts towards ischemia-reperfusion injury
Source: Front Genet. 2022 Nov 15;13:995887. doi: 10.3389/fgene.2022.995887 (PMC9705337; doi:10.3389/fgene.2022.995887)
Supplement: Supplementary file 1 [file Table1.DOCX]

**Supplementary table**

| **S.No.** | **Gene** | **Forward primer** | **Reverse primer** |
| --- | --- | --- | --- |
| 1 | GAPDH | 5'-GCGAGATCCCGCTAACATCA-3' | 5'-CTCGTGGTTCACACCCATCA-3' |
| 2 | PGC 1α | 5'-GAGGGACGAATACCGCAGAG-3' | 5'-CTCTCAGTTCTGTCCGCGTT-3' |
| 3 | Dnm1 | 5'-TTGCCCTCTTCAACACTGAGC-3' | 5'-ATGAAGCTGTCAGAGCCGTT-3' |
| 4 | Parkin | 5'-AGTTTGTCCACGACGCTCAA-3' | 5'-CAGAAAACGAACCCACAGCC-3' |
| 5 | MFN1 | 5'-TGACTTGGACTACTCGTGCG-3' | 5'-GGCACAGTCGAGCAAAAGTG-3' |
| 6 | MFN2 | 5'-CTCTGTGCTGGTTGACGAGT-3' | 5'-TCGAGGGACCAGCATGTCTA-3' |
| 7 | DRP1 | 5'-TGGAAAGAGCTCAGTGCTGG-3' | 5'-TCAACTCCATTTTCTTCTCCTGT-3' |
| 8 | MFF | 5'-GAAAACACCTCCACGTGTGC-3' | 5'-CTGCTCGGATCTCTTCGCTT-3' |
| 9 | FIS | 5'-CCAGAGATGAAGCTGCAAGGA-3' | 5'-TTCCTTGAGCCGGTAGTTGC-3' |
| 10 | PINK1 | 5'-TGTATGAAGCCACCATGCCC-3' | 5'-TCTGCTCCCTTTGAGACGAC-3' |
| 11 | TFAM | 5'-GTTGCTGTCGCTTGTGAGTG-3' | 5'-GTCTTTGAGTCCCCCATCCC-3' |
| 12 | β-actin | 5'-GTGTGGTCAGCCCTGTAGTT-3' | 5'-CCTAGAAGCATTTGCGGTGC-3' |
| 13 | POLG1 | 5'-CTTTGGGCTCCAGCTTGACT-3' | 5'-TGGAGAAAATGCTTGGCACG-3' |
| 14 | ND1 | 5'-CCACCGCGGTCATACGATTA-3' | 5'-AGGGCTAAGCATAGTGGGGT-3' |
| 15 | CYTB | 5'-ACAAAATCCCATTCCATCCA-3' | 5'-GTTGGGAATGGAGCGTAGAA-3' |
| 16 | ND6 | 5'- ATCCGGAAACTTGAGGGTCT-3' | 5'-CCCAGCCACCACTATCATTC-3' |
| 17 | ND5 | 5'- ATTGCAGCCACAGGAAAATC-3' | 5'-TGGTGATTGCACCAAGACAT-3' |
| 18 | ND4L | 5'-GGTACTTTTATATTTCGCTCCCACT-3' | 5'-CGCAGGCTGCAAAAACTAGA-3' |
| 19 | ND3 | 5'-TGCATTCTGATTGCCTCAAA-3' | 5'-TGGGAGGGGGAGTAGTAAGG-3' |
| 20 | COX3 | 5'-AGCCCATGACCACTAACAGG-3' | 5'-TGGCCTTGGTATGTTCCTTC-3' |
| 21 | ATP6 | 5'-ACACCAAAAGGACGAACCTG-3' | 5'-AGAATTACGGCTCCTGCTCA-3' |
| 22 | ATP8 | 5'-ACACCAAAAGGACGAACCTG-3' | 5'-AGAATTACGGCTCCTGCTCA-3' |
| 23 | COX2 | 5'-GCTTACAAGACGCCACATCA-3' | 5'-GAATTCGTAGGGAGGGAAGG-3' |
| 24 | COX1 | 5'-AATTGGAGGCTTCGGAAACT-3' | 5'-CTGTTCCAGCTCCAGCTTCT-3' |
| 25 | ND2 | 5'-AAAAAGCCCACGATCAACTG-3' | 5'-GGGAATTCCTTGGGTGACTT-3' |
| 26 | ND4 | 5'-CCCACTCTTAATTGCCCTCA-3' | 5'-CGTGGGCTTTTGGTAATCAT-3' |
| 27 | DNMT1 | 5’CGGATTGTCGGATAAAAGA3’ | 5’GCTTCCTCATCGCTCCAGTA3’ |
| 28 | DNMT 3A | 5′-GGAGAGGAAAGGGAGAGAGG -3′ | 5′-AGGGATGGTGCTGGTGAGAC-3′ |
| 29 | DNMT3B | 5′-AAACCCAACAACAAGCAACC-3′ | 5′-ACATCAGAAGCCATCCGTTC -3’ |
| 30 | TET1 | 5′-TATATGGCTGTGCTGTGCTGCCCAA-3′ | 5′-CGATGGGCCATTGCTTGATG -3′ |
| 31 | TET2 | 5′-TGTTGTCAGGGTGAGAATCCAG-3’ | 5′-CCTGTAGGCATCAGGTGCAA -3′ |
| 32 | TET3 | 5′-CCCTTGCCTGAAGCATCTCA -3′ | 5′-GCCGAGGTACCATTCCCAAA -3′ |

**Supplementary table 1. Primer sequence details:** The forward and reverse primer sequences of the genes used for real time PCR analysis are presented
